# Superior Mesenteric Embolism—Rapid Gangrene of Intestine

**Published:** 1918-09

**Authors:** 


**Superior Mesenteric Embolism—Rapid Gangrene of Intestine.** Guibe and Moreau, Le Prog. Med., Feb. 9, cites a case following toxic appendicitis.

